# Artemisinin and Its Derivatives: Promising Therapeutic Agents for Age-Related Macular Degeneration

**DOI:** 10.3390/ph18040535

**Published:** 2025-04-06

**Authors:** Chun Liu, Xiaoqin Liu, Junguo Duan

**Affiliations:** 1 Eye School, Chengdu University of TCM, Chengdu 610075, China; 2 Clinical Medical School, Chengdu University of TCM, Chengdu 610075, China; 3 Key Laboratory of Sichuan Province Ophthalmopathy Prevention & Cure and Visual Function Protection with TCM Laboratory, Chengdu 610075, China

**Keywords:** artemisinin, dihydroartemisinin, artesunate, artemether, age-related macular degeneration

## Abstract

Age-related macular degeneration (AMD) is a leading cause of visual impairment and blindness in older adults. Its pathogenesis involves multiple factors, including aging, environmental influences, genetic predisposition, oxidative stress, metabolic dysfunction, and immune dysregulation. Currently, AMD treatment focuses primarily on wet AMD, managed through repeated intravitreal injections of anti-vascular endothelial growth factor (VEGF) therapies. While anti-VEGF agents represent a major breakthrough in wet AMD care, repeated injections may lead to incomplete responses or resistance in some patients, and carry a risk of progressive fibrosis. Artemisinin (ART) and its derivatives, originally developed as antimalarial drugs, exhibit a broad spectrum of pleiotropic activities beyond their established use, including anti-inflammatory, anti-angiogenic, antioxidant, anti-fibrotic, mitochondrial regulatory, lipid metabolic, and immunosuppressive effects. These properties position ART as a promising therapeutic candidate for AMD. A growing interest in ART-based therapies for AMD has emerged in recent years, with numerous studies demonstrating their potential benefits. However, no comprehensive review has systematically summarized the specific roles of ART and its derivatives in AMD pathogenesis and treatment. This paper aims to fill the knowledge gap by synthesizing the therapeutic efficacy and molecular mechanisms of ART and its derivatives in AMD, thereby providing a foundation for future investigations.

## 1. Introduction

Age-related macular degeneration (AMD) represents a leading cause of vision impairment in developed nations, resulting in irreversible vision loss among individuals aged 60 years and older [1]. This condition contributes to 8.7% of global blindness cases [2]. Current epidemiological projections suggest the number of AMD patients will reach 288 million by 2040, driven by increasing life expectancy and the global adoption of Western dietary patterns and sedentary lifestyles [3]. AMD manifests as a chronic neurodegenerative condition affecting retinal photoreceptors, retinal pigment epithelium (RPE), and Bruch’s membrane within the macular region [4]. Clinically, it presents in two forms: dry (atrophic) and wet/neovascular AMD (wAMD/nvAMD) [5]. The dry form constitutes approximately 85% of cases, characterized by progressive RPE dysfunction accompanied by photoreceptor degeneration. Despite its prevalence, the pathogenesis of dry AMD remains elusive, with no approved disease-modifying therapies currently available. In contrast, wAMD, representing 10–15% of AMD cases, accounts for over 90% of severe vision loss [1,6]. This neovascular variant is pathologically defined by choroidal neovascularization (CNV), wherein abnormal choroidal vessels invade the subretinal space, triggering macular edema, exudates, hemorrhages, and fibrosis [7].

The current standard of care for wAMD involves anti-vascular endothelial growth factor (VEGF) agents [8,9]. Nevertheless, this therapeutic approach faces several limitations, including the requirement for frequent intravitreal injections [10], risks of endophthalmitis and fibrotic complications, along with suboptimal or non-existent responses in a subset of patients [11,12]. Additionally, anti-VEGF therapies entail high costs and transient therapeutic effects [10]. The combined clinical and socioeconomic impacts of AMD significantly compromise patients’ quality of life while imposing substantial economic burdens, establishing AMD as a critical global public health priority. These challenges underscore the urgent need for cost-effective, minimally invasive therapies with sustained efficacy.

Traditional Chinese Medicine has emerged as a promising source for novel therapeutics. The Nobel Prize-winning discovery of artemisinin (ART) by Youyou Tu—an antimalarial compound isolated from Artemisia annua—has catalyzed investigations into its broader pharmacological applications [13,14,15]. Emerging preclinical evidence suggests ART and its derivatives may exhibit multi-target therapeutic mechanisms relevant to AMD management [16,17]. Despite growing research interest, no comprehensive synthesis exists regarding ART’s specific therapeutic potential in AMD pathogenesis. This review systematically examines the emerging roles of ART and its analogs in AMD treatment through multiple mechanistic perspectives, providing a foundation for future therapeutic development.

## 2. Overview of ART and Its Derivatives

**Figure 1:**
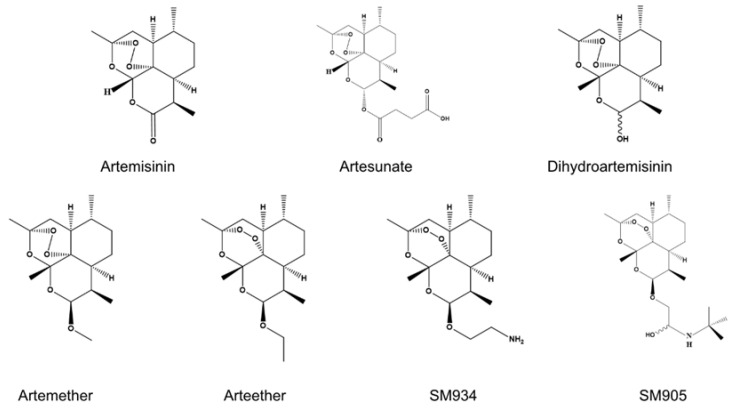
Chemical structures of artemisinin and several representative derivatives.

ART is a natural sesquiterpene lactone containing a peroxy bridge structure, isolated from *Artemisia annua* L., a plant of the genus Artemisia [18]. However, its therapeutic efficacy is limited by low solubility in both oil and water, poor oral bioavailability, and a short plasma half-life [19,20]. To address these limitations, researchers have synthesized structurally modified derivatives of ART, including artesunate (ARTS), dihydroartemisinin (DHA), artemether, SM934, and SM905 [15] (Figure 1). These derivatives exhibit optimized pharmacokinetic properties, such as accelerated absorption, enhanced tissue distribution, efficient systemic clearance, and reduced plasma concentrations, resulting in improved clinical efficacy and stability as antimalarial agents [21]. Furthermore, studies have demonstrated that ART and its derivatives possess broad pharmacological activities, including anti-inflammatory, anti-angiogenic, antioxidant, anti-fibrotic, and immunomodulatory effects [22,23,24,25]. Further exploration of their therapeutic potential in chronic and refractory diseases may expand their clinical applications.

With the broadening of their clinical applications, safety evaluation has become a critical factor in clinical decision-making. Preclinical and clinical evidence indicates that ART and its derivatives generally demonstrate a favorable safety profile; however, risk assessment remains essential in specific therapeutic contexts [26]. A systematic review of 77 clinical studies reported adverse events (AEs) in 59 trials, with only one study documenting a Grade 3 AE that did not necessitate treatment discontinuation. The remaining studies showed no severe drug-related reactions [27]. In trials involving healthy volunteers, ARTS injections (intravenous or intramuscular) caused no serious AEs. All reported AEs were mild and self-limiting, including transient bitter taste (intravenous), injection site pain (intramuscular), headaches, and nausea [28]. Electrocardiogram monitoring detected no cardiac toxicity, such as QT interval prolongation, and laboratory parameters remained within normal ranges.

Despite this evidence, several key issues warrant attention. First, dose-dependent toxicity must be considered, as elevated intracellular drug concentrations can induce concentration-dependent cytotoxicity [29,30]. Additionally, prolonged low-dose exposure may compromise drug stability due to free radical scavenger accumulation [31]. Second, variations in tissue drug concentrations across pathological conditions highlight the need for disease-specific dose–response models [19].

In summary, artemisinin-based drugs demonstrate a favorable safety profile under standardized regimens, with mild, self-limiting adverse reactions (e.g., transient liver enzyme elevation and gastrointestinal symptoms). However, expanding their clinical use requires rigorous evaluation of long-term risks, tissue-specific toxicity, and drug interactions.

## 3. Role of ART and Its Derivatives in AMD

AMD is a disease that primarily disrupts the RPE, photoreceptor cells, and the choriocapillaris-Bruch’s membrane complex. The etiology and pathogenesis of AMD remain unclear, although age is undoubtedly the primary risk factor. Genome-wide studies reveal over 60 susceptibility loci, with CFH (chromosome 1q32) and ARMS2/HTRA1 (10q26) as core risk variants driving complement dysregulation, chronic inflammation, and retinal damage [32]. Polymorphisms in lipid metabolism (APOE, ABCA1, CETP) and extracellular matrix genes (TIMP3, MMP) exacerbate lipid deposition and pathological remodeling [33]. Complement genes (C3, CFB, CFI) further contribute to immune activation, while gene-environment interactions shape AMD progression [34]. Additionally, there is evidence to suggest that smoking, gender, race, obesity, and sunlight exposure may also play a role [35,36,37].

The principal pathogenic mechanisms of AMD include oxidative stress, choroidal vascular dysfunction, inflammation, aberrant lipid metabolism, and immune dysregulation [38,39,40]. RPE cells in AMD patients are particularly susceptible to oxidative damage, mitochondrial dysfunction, aberrant apoptosis, and age-related geographic atrophy, leading to impaired cellular function [41,42]. Inflammatory and metabolic abnormalities can alter Bruch’s membrane permeability, disrupting nutrient transport and contributing to atrophy of the RPE, Bruch’s membrane, and choroidal capillaries [43]. Collagen thickening in Bruch’s membrane may rupture its elastic lamina, allowing choroidal capillaries to infiltrate the sub-pigmented or sub-neuroepithelial layers through membrane fissures, thereby initiating CNV lesions [44]. CNV proliferation and hemorrhage promote fibrovascular membrane formation via extracellular matrix remodeling, culminating in connective tissue deposition [45]. Late-stage scar tissue formation disrupts retinal and choroidal architecture, ultimately causing irreversible central vision loss [46]. ART and its derivatives modulate critical pathways—such as inflammation, angiogenesis, oxidative stress, fibrosis, mitochondrial homeostasis, lipid metabolism, and immune responses—thereby mitigating AMD-associated pathological damage under diverse stimuli [16,47]. As shown in Table 1, the existing literature suggests that ART and its derivatives may affect AMD.

### 3.1. ART and Its Derivatives Inhibit Inflammation

Inflammation is hypothesized to play a central role in the pathogenesis of both dry and neovascular (wet) AMD. In dry AMD, photoreceptor and RPE cell damage arises primarily from lipofuscin accumulation and impaired lysosomal enzyme phagocytic activity. Proinflammatory mediators such as TNF-α, IFNγ, and IL-1β trigger RPE cells to release cytokines and chemokines, leading to chronic inflammation that disrupts the blood–retinal barrier through increased vascular permeability [54,55,56]. In wAMD, proinflammatory mediators drive CNV, a hallmark feature linked to inflammatory cytokine release, complement system activation, and macrophage/microglia regulation [57,58]. CNV lesions are inherently unstable; localized inflammation and persistent immune activation exacerbate aberrant neovascularization, causing retinal edema, hemorrhage, and progressive vision loss [59]. Thus, inflammatory mechanisms underlie both degenerative processes and complications in AMD.

The NF-κB transcription factor family regulates immune, inflammatory, proliferative, and apoptotic responses, with additional roles in tumorigenesis and cell migration [60,61]. Under physiological conditions, NF-κB remains inactive in the cytoplasm. Pathological stimuli trigger nuclear translocation of NF-κB dimers, activating transcription of proinflammatory genes (e.g., TNF-α, MMP9, COX-2, IFNγ, IL-6) [62]. ART and its derivatives inhibit NF-κB signaling by blocking cytoplasmic-nuclear translocation. For instance, ART reduced NF-κB p65 nuclear translocation in Aβ1-42-stimulated BV2 microglia by suppressing TLR4/NF-κB activation, thereby lowering TNF-α, IL-1β, and IL-6 levels [63]. This mechanism involves stabilization of I-κBα, as shown in PMA-induced THP-1 monocytes, where ART inhibited IκBα phosphorylation and degradation for up to 6 h [64]. Similarly, ART decreased TNF-α-induced NO, PGE₂, and COX-2 production via NF-κB pathway inhibition [65]. ARTS and DHA also suppress NF-κB-mediated gene transcription and inflammatory responses [66,67], while SM905 exhibits anti-inflammatory effects through dual inhibition of MAPK and NF-κB pathways [68].

MAPK signaling—encompassing ERK, JNK, and p38 subtypes—synergizes with NF-κB to amplify inflammatory gene expression [69]. p38 MAPK is particularly critical in AMD pathogenesis: its activity increases with age in healthy retinas and is hyperactivated in AMD models, correlating with CryaB phosphorylation and disease progression [70]. Senescent RPE cells promote angiogenesis via the TAK1/p38 pathway [71], suggesting p38 inhibition as a therapeutic strategy. Experimental data show ARTS suppresses ERK, JNK, and p38 phosphorylation, reducing inflammatory cytokine production [72]. ART also inhibits TNF-α-induced p38 phosphorylation in HUVECs, downregulating ICAM-1 and VCAM-1 to attenuate leukocyte adhesion [73]. These findings align with studies demonstrating ART’s coordinated suppression of NF-κB and MAPK pathways [74].

The NLRP3 inflammasome—a key component of innate immunity—initiates inflammatory responses upon pathogen or damage recognition. ART and its derivatives modulate NLRP3 activation: ART downregulates NLRP3, ASC, caspase-1, and IL-1β [75], while DHA inhibits NLRP3 inflammasome assembly and proinflammatory cytokine release [76]. SM934, a water-soluble ART derivative, curtailed the accumulation of TLR4 by regulating the TLR4/NF-κB/NLRP3 signaling pathway, thereby inhibiting its activation and significantly reducing the levels of inflammatory mediators TNF-α, IL-6, IL-10, and IL-1β [77]. Targeting these inflammatory pathways with ART-based therapies may offer novel approaches for AMD management.

### 3.2. ART and Its Derivatives Against Neovascularization

CNV refers to the formation of abnormal new blood vessels in the choroid. Penetration of these vessels through Bruch’s membrane is a major contributor to vision loss in AMD patients. CNV formation is typically triggered by retinal or choroidal injury, which induces RPE cells to release inflammatory mediators and pro-angiogenic factors, such as VEGF, angiopoietin, FGF, and TGF. These factors regulate endothelial cell behavior in vitro [78], with VEGF serving as the primary driver [79]. VEGF, a paracrine/autocrine signaling protein secreted by macrophages, RPE cells, endothelial cells, and pericytes [80,81,82], exerts dual roles in physiological and pathological contexts. Under normoxic conditions, VEGF supports bone development, hematopoiesis, wound healing, and vascular/lymphatic system formation [83,84]. In contrast, hypoxia or inflammatory microenvironments induce pathological VEGF overexpression, increasing vascular permeability, degrading extracellular matrix (ECM), and promoting endothelial cell migration and proliferation—key steps in angiogenesis [85,86].

Hypoxia-inducible factor 1α (HIF-1α) is central to VEGF upregulation. During hypoxia, HIF-1α escapes proteasomal degradation, accumulates intracellularly, and translocates to the nucleus, where it binds hypoxia-response elements in the VEGF promoter to enhance transcription [87]. Concurrently, ECM remodeling—including collagen and glycosaminoglycan synthesis/reorganization—provides structural support for nascent vessels. Dynamic cell-ECM interactions further facilitate neovascularization by modulating endothelial cell adhesion and signaling.

The reduction in CNV formation and growth represents a significant objective in the management of wAMD. ART and its derivatives exhibit antiangiogenic properties by downregulating pro-angiogenic factors (e.g., VEGF, FGF, HIF-1α, Ang-1) while upregulating angiogenesis inhibitors and reducing VEGFR-1/VEGFR-2 expression [88,89,90].

In vitro, DHA suppresses VEGF and inhibits endothelial cell proliferation, migration, and tube formation—key steps in angiogenesis [91]. Mechanistically, DHA reduces VEGFR-2 mRNA/protein levels by blocking NF-κB p65 nuclear translocation, thereby suppressing VEGFR-2 promoter activity [92]. It further attenuates angiogenesis via mTOR and HIF-1α downregulation [93]. In an experimental laser-induced CNV mouse model, DHA demonstrated notable anti-angiogenic effects. When administered orally (via intragastric gavage), DHA was observed to significantly reduce the expression of VEGF and its receptor VEGFR-2, as well as inhibit the NF-κB signaling pathway. This resulted in a notable reduction in edema and the area of CNV without any appreciable toxicity to the retina [48].

ARTS, another ART derivative, inhibits ocular neovascularization by downregulating VEGFR-2, PKCα, and PDGFR. Compared to bevacizumab, ARTS showed superior ocular penetration and longer-lasting effects in rabbit/monkey models, highlighting its potential as a small-molecule therapy for ocular neovascularization and suggesting its multi-target action may overcome limitations of anti-VEGF drugs and their side effects [50]. Both DHA and ARTS dose-dependently suppressed angiogenesis in human umbilical vein endothelial cell assays (12.5–50 μM for DHA; 2.5–50 μM for ARTS), with DHA demonstrating greater potency [94].

Pathologically elevated VEGF disrupts the blood–retinal barrier, increasing vascular permeability and macular exudation—key drivers of vision-threatening retinal ischemia [95,96]. Notably, a single 20 μg ARTS injection reversed neovascularization, vascular abnormalities, and fluorescein leakage in rabbit/monkey models for six months [50], highlighting its translational potential for wAMD.

### 3.3. ART and Its Derivatives Inhibit Oxidative Stress

A substantial amount of energy is required for the retina to convert light into visual signals, during which reactive oxygen species (ROS) are generated as by-products of normal metabolic processes [97]. ROS are produced during oxidative metabolism and participate in cellular signaling [98]. However, when ROS generation exceeds the capacity of antioxidant defenses (e.g., superoxide dismutase, catalase), redox balance is disrupted, leading to oxidative stress [99]. This damages cell membranes, lipids, proteins, and DNA, impairing their normal functions. Retinal function critically depends on oxygen-dependent metabolism [100]. Factors such as age-related declines in antioxidant enzyme activity increase ROS levels—including superoxide radicals (·O₂⁻), hydroxyl radicals (·OH), and hydrogen peroxide (H₂O₂)—thereby elevating retinal oxidative damage risk [101].

Recent studies confirm that oxidative stress is central to AMD pathogenesis [43,102], an age-related disorder characterized by cumulative cellular replication that shortens telomeres and reduces proliferative capacity, particularly in RPE cells. This induces replicative senescence and lysosomal dysfunction in RPE cells, causing accumulation of intracellular waste like lipofuscin—a light-sensitive pro-oxidant that generates ROS and exacerbates lysosomal defects and phagocytic impairment [103,104]. Such vicious cycles accelerate AMD progression [105]. Additionally, mitochondrial DNA damage and impaired waste clearance promote Bruch’s membrane thickening, disrupting RPE-photoreceptor interactions [106,107].

ART and its derivatives demonstrate therapeutic potential through multi-target antioxidant actions. The ERK/CREB pathway—a master regulator of proliferation and survival [108]—is modulated by ART to protect retinal cells. Yan et al. showed ART rescues H_2_O_2_-damaged RGC-5 cells via ERK1/2 and p38 pathway regulation, restoring electroretinogram a/b-wave amplitudes in light-injured rats [53]. Electroretinogram analysis in light-injured rats showed ART restored a/b-wave amplitudes in a concentration-dependent manner, demonstrating neuroprotection against oxidative damage. Chong et al. observed similar effects in human D407 RPE cells, where ART normalized nuclear morphology, reduced ROS levels, and attenuated H₂O₂-induced apoptosis [17]. Li et al. identified an AMPK-mediated mechanism: ART reduced mitochondrial ROS, prevented membrane potential collapse, and mitigated lactate dehydrogenase release in primary RPE cells—effects abolished by AMPK inhibitors [51]. Yang et al. further showed ART activates the CaMKK2/AMPK/Nrf2 axis, enhancing antioxidant gene expression and modulating histone acetylation to reduce H₂O₂-induced RPE apoptosis [52,109]. These findings validate ART’s capacity to maintain oxidative homeostasis in high-risk retinal microenvironments [110,111], positioning it as a promising AMD therapeutic candidate.

### 3.4. ART and Its Derivatives Against Fibrosis

Fibrosis represents a maladaptive pathological response characterized by excessive ECM accumulation and the replacement of functional parenchyma with nonfunctional fibrous tissue [112]. While this reparative mechanism helps preserve structural continuity, the resultant scar tissue lacks the specialized functionality of native parenchyma. This dysregulated process proves particularly detrimental in organs requiring precise architectural organization and dynamic cellular interactions, where even localized fibrotic lesions can precipitate systemic organ dysfunction [113]. Subretinal fibrosis, a hallmark of advanced AMD, manifests through scar formation that disrupts photoreceptors, RPE, and choroidal capillaries, culminating in irreversible central vision loss [114]. The exact pathogenesis of subretinal fibrosis remains incompletely understood. Current models propose that in wAMD, fibrosis emerges as an aberrant resolution of chronic tissue repair, progressing through three overlapping phases: inflammatory activation, myofibroblast-driven ECM deposition, and failed tissue remodeling. Following retinal injury, epithelial cells initiate mediator release to recruit inflammatory cells, endothelial cells, and fibroblasts, while simultaneously undergoing epithelial–mesenchymal transition to generate myofibroblasts [115,116].

Although anti-VEGF therapy achieves visual improvement in most wAMD patients [117], long-term studies reveal approximately an experienced 25% substantial vision decline (defined as ≥15-letter decrease on ETDRS charts) despite decade-long treatment [118]. This clinical deterioration correlates strongly with subretinal fibrosis development in 50% of anti-VEGF-treated wAMD cases. Notably, no approved anti-fibrotic therapies currently exist for this indication. The fibrotic cascade in wAMD initiates through neovascular leakage that establishes chronic tissue damage and inflammatory mediator-rich microenvironments. Subsequent myofibroblast activation and dysregulated ECM remodeling drive fibrovascular complex formation—a pathological endpoint characterized by vascular fibrotic conversion and permanent macular damage [119].

The anti-fibrotic properties of ART and its derivatives stem from their multimodal regulation of key signaling cascades. Beyond modulating initial inflammatory responses, these compounds demonstrate therapeutic potential through coordinated interference with TGF-β, MAPK, PI3K/AKT, and other fibrosis-associated pathways [120]. As a central mediator of fibrogenesis, TGF-β activates both canonical and non-canonical signaling routes [121,122]. In the canonical pathway, TGF-β ligand binding induces TGFBR2-mediated phosphorylation of TGFBR1, forming an active receptor complex that catalyzes Smad2/3 C-terminal phosphorylation. The resulting Smad2/3-Smad4 complex translocates to the SBEs on profibrotic gene promoters [123,124]. Concurrently, TGF-β activates non-canonical effectors including MAPK (p38/JNK), PI3K/AKT, and Rho GTPase pathways that orchestrate proliferative, migratory, and differentiation responses contributing to fibrotic progression [125]. Experimental evidence provides confirmation that ART and its derivatives are involved in mediating pathway modulation: Zheng et al. reported DHA suppresses TGF-β expression and TGF-β1/Smad2/3 signaling, significantly reducing ECM deposition in fibrotic models [126]. Similarly, Nong et al. observed ARTS inhibit TGF-β1/Smad3 signaling to diminish fibroblast activity and collagen synthesis, effectively mitigating scar formation [127]. Li et al. further demonstrated ARTS attenuate Smad2/3 phosphorylation via p38/JNK inhibition, thereby reducing cellular activation states [128]. Complementing these findings, Wang et al. established ARTS-mediated PI3K/Akt downregulation suppresses TGF-β2-induced RPE cell migration and epithelial–mesenchymal transition [129].

The anti-fibrotic mechanisms of ART and its derivatives involve comprehensive ECM remodeling. These compounds consistently reduce α-smooth muscle actin (α-SMA), collagen, and MMP expression while upregulating tissue inhibitors of metalloproteinases. Wang et al. documented ARTS-induced decreases in collagen-IV along with increased MMP-2/9 and TIMP-1/2 expression, effectively counteracting ECM accumulation in pulmonary fibrosis [130]. This regulatory pattern was corroborated by Xu et al. in hepatic fibrosis models, where ARTS suppressed MMP-2/9, α-SMA, and collagen I expression [131]. At the cellular level, Lv et al., revealed ARTS-mediated FAK/Akt/β-catenin inhibition reduces α-SMA and collagen I mRNA in hepatic stellate cells, significantly limiting ECM production [132]. Notably, Liu’s research indicated that ARTS could inhibit the TGF-β1/SMAD2/3 and PI3K/Akt signaling pathways in primary human ocular fibroblasts, leading to mitochondrial-dependent ferroptosis in fibroblasts. This process is characterized by mitochondrial dysfunction, mitochondrial fission, and iron-dependent lipid peroxidation, which significantly inhibit fibroblast activation and promote cell death, ultimately contributing to the attenuation of fibrosis [133]. In ocular angiogenesis models, ARTS treatment markedly attenuated choroidal endothelial cell migration and inflammatory/fibrotic factor expression, while choroid/RPE co-cultures showed reduced fibrovascular growth [47]. Laser-induced CNV models confirmed ARTS capacity to inhibit both neovascularization and fibrosis formation. Mechanistically, Larson et al. demonstrated ART-mediated downregulation of myofibroblast markers and ECM genes in dermal fibroblasts, coupled with pro-apoptotic effects [134]. In summary, ART and its derivatives have been shown to exert significant anti-fibrotic effects by inhibiting pro-fibrotic signaling pathways and preventing the excessive production of pathological myofibroblasts. This provides optimistic rationale for their potential as anti-fibrotic drugs for AMD.

### 3.5. ART and Its Derivatives Maintain Mitochondrial Homeostasis

It is well established that the retina is an area of the human body with a markedly elevated oxygen consumption and metabolic rate [97]. This high-energy demand correlates with the abundant mitochondria populating RPE cells and their specialized membrane ion channels [135,136]. As cellular powerplants, mitochondria are essential for maintaining RPE physiological functions. Notably, the mitochondrial electron transport chain constitutes a major ROS source in human RPE systems [137,138,139]. It is imperative to acknowledge the significance of mitochondrial homeostasis in the context of normal cellular biological processes. Nevertheless, a decline in mitochondrial function over time may result in the release of deleterious ROS and DNA, which in turn triggers oxidative stress, inflammation, and cellular damage. These processes are closely associated with the manifestation of a wide range of age-related diseases [140]. In recent years, there has been a growing body of research examining the role of mitochondrial homeostasis in the development of AMD [141]. Mitochondrial dysfunction is regarded as a significant contributor to the pathogenesis of the disease [138,142].

Table 2 summarizes the role of mitochondrial dysregulation in the pathogenesis of AMD and outlines the potential therapeutic modulation by ART and its derivatives. Experimental studies demonstrate ART effectively restores nuclear morphology and reduces intracellular ROS in concentration-dependent fashion, while preserving mitochondrial membrane potential and suppressing caspase-3 activation, thereby mitigating oxidative stress-induced mitochondrial damage [17,143]. Mitochondria are not static structures; rather, they are constantly undergoing fusion and fission. Qin et al. revealed that ARTS not only reduce mitochondrial ROS production but also regulate fusion-fission balance, promoting mitochondrial network remodeling [144]. Mechanistically, ART preserves mitochondrial integrity through AMPK activation-mediated membrane potential stabilization [51], while artemether enhances mitochondrial resilience via pyruvate metabolism regulation and the enhancement of biogenesis [145].

The mitochondrial biogenesis regulator PGC-1α plays pivotal roles in oxidative phosphorylation and antioxidant defense, critical for RPE protection [146]. Intriguingly, artemether elevates mitochondrial hydrogen peroxide flux while upregulating PGC-1α expression and downregulating pyruvate dehydrogenase kinase 1, effectively rebalancing redox homeostasis [147]. Supporting these findings, Han et al. reported artemether attenuates tissue damage through dual redox equilibrium restoration and mitochondrial function improvement [148]. It may, therefore, be concluded that maintenance and regulation of mitochondrial function represent an effective target for the prevention and treatment of AMD [138].

### 3.6. ART and Its Derivatives Regulate Lipid Metabolism

Lipid accumulation represents a pathological manifestation of dysregulated lipid homeostasis, with drusen formation—lipid-rich deposits between RPE and Bruch’s membrane—serving as hallmark features of dry AMD [149,150]. Although clinically asymptomatic, drusen constitute important biomarkers for AMD progression [151]. Emerging evidence implicates lipid metabolism in AMD pathogenesis, supported by population studies linking high saturated fat/cholesterol intake with elevated early AMD risk [152]. Pathological lipid accumulation within RPE and basement membranes drives macular ROS elevation, triggering lipid peroxidation, mitochondrial dysfunction, and oxidative retinal damage [153,154]. Notably, omega-3 fatty acids exhibit inverse correlations with AMD incidence, while omega-6/omega-3 ratio may influence disease development [155]. Epidemiological data further suggest ω-3 PUFA-rich diets and fish consumption associate with reduced AMD risk [156]. Contrasting conventional paradigms, Wang et al. reported positive correlations between HDL levels and AMD risk, while elevated total cholesterol, LDL, and triglycerides (TG) showed paradoxical protective associations [157]. These findings align with Colin et al.’s experimental data confirming HDL’s positive and TG’s negative correlations with early AMD and vitelliform lesions [158]. Current therapeutic strategies targeting lipid metabolism include docosahexaenoic acid, desipramine, apolipoprotein mimetics, and statins [159].

Experimental evidence demonstrates ART and its derivatives’ multifaceted lipid-modulating capabilities. At 5 μM, ARTS significantly inhibits lipid accumulation and triglyceride synthesis during 3T3-L1 adipocyte differentiation, concurrently suppressing PPAR-γ and fatty acid synthase expression [160]. Similarly, DHA concentration- and time-dependently reduces adipogenic differentiation via downregulation of CEBPA, PPARG, FABP4, and PLIN [161]. Mechanistically, ART analogs impair lipid storage through IGF1R-PI3K-AKT pathway inhibition, reducing adipogenic markers (PLIN1, PPARG) [162]. Beyond anti-lipogenic effects, ARTS enhance lipolysis through adipocyte lipase activation, promoting fatty acid release and oxidative metabolism. Notably, ART upregulates lipoprotein lipase via KLF2/NRF2/TCF7L2 signaling, enhancing triglyceride hydrolysis [163].

Another mechanism by which it regulates lipid metabolism is through its influence on cholesterol metabolism. Studies have demonstrated that ARTS promotes β-oxidation of fatty acids, inhibits fatty acid synthesis, facilitates the conversion of cholesterol to bile acids, and decreases the levels of total cholesterol (TC), TG and LDL, while increasing the levels of HDL [164]. ARTS is able to regulate lipid metabolism by inhibiting the ERK1/2/NF-κB/IL-1β pathway, increasing HDL levels and decreasing LDL expression, thereby regulating lipid metabolism [165]. Experimental validation shows artemether effectively reduces serum TC and LDL [166], while DHA demonstrates similar lipid-lowering capacity [167]. These multimodal lipid-regulatory properties position ART and its derivatives as promising candidates for AMD progression mitigation.

### 3.7. ART and Its Derivatives Modulate Immunity

The blood–retinal barrier comprises two principal components: retinal vascular endothelial cells interconnected by tight junctions and RPE [168]. This barrier function maintains ocular immune privilege by restricting inflammatory cell/protein influx, thereby minimizing inflammatory damage. However, this immunoregulatory state may impair retinal self-antigen tolerance, enabling blood–retinal barrier compromise under pathological conditions to expose retinal autoantigens and initiate autoimmune responses via immune cell infiltration [169]. Accumulating evidence implicates immune dysregulation in AMD pathogenesis, with autoantibodies specifically targeting retinal components observed in 94% of early AMD cases and 83% of advanced AMD patients versus 9% in controls [170,171,172]. Disease progression correlates with retinal tissue-specific IgG response patterns [173], while age-related microglial dysfunction and impaired phagocytosis further contribute to AMD development [174]. Experimental models confirm T-cell and B-cell involvement in AMD pathology [175], supported by single-cell RNA sequencing revealing CD16+ monocyte signatures associated with anti-VEGF treatment burden [176]. This finding provides a crucial foundation for elucidating the relationship between AMD and the immune system and for the development of novel therapies.

ART and its derivatives exhibit broad-spectrum immunomodulatory properties through immune cell regulation (neutrophils, macrophages, dendritic cells, T-cells) [177]. It has been demonstrated that ART enhances the capacity of these immune cells to eliminate pathological alterations and facilitate the process of tissue repair by modifying their polarization status. This mechanism is of great significance in the prevention and treatment of AMD. ART has been demonstrated to possess the capacity to selectively inhibit neutrophil and macrophage chemotaxis, while concomitantly decreasing the release of cytokines, chemokines, and exogenous NETs. These findings illustrate the potential therapeutic value of ART [178]. Zhao et al. observed that ART significantly inhibited microglia activation and reduced the production of NO, ROS, and a variety of inflammatory factors. This resulted in a reduction in the migration ability of microglia [63]. In addition, DHA regulates the function of the immune system by adjusting the number of regulatory T cells and their differentiation [179]. ARTS was found to have strong immunosuppressive activity, inhibiting the proliferation of T lymphocytes induced by allergens and other antigens, and significantly suppressing delayed hypersensitivity reactions. Additionally, ARTS was observed to facilitate the proliferation of regulatory T cells [180]. In the laser-induced CNV model, ARTS was observed to significantly reduce the recruitment and infiltration of mononuclear phagocytes, which was closely related to its effect on reducing CNV and scar formation. Furthermore, this effect was corroborated by the reduced expression of proinflammatory and pro-fibrotic factors in choroidal endothelial cells following incubation with ARTS [47]. DHA was demonstrated to inhibit the induction of Tfh cells and their paracrine differentiation of B cells, which directly inhibits B cell activity and reduces the production of pathogenic antibodies. This provides promising results for the treatment of autoimmune diseases [181]. Notably, SM934 demonstrates 35-fold greater lymphocyte proliferation inhibition than DHA, potently suppressing Th17 generation and Th1 polarization [182,183]. Mechanistically, ARTS restores Th17/Treg balance through metabolic reprogramming of CD4+ T-cells (glycolysis, lipid/amino acid metabolism) [184], while DHA coordinates Th1/Th2 cytokine equilibrium [67]. These multimodal immunoregulatory effects position ART and its derivatives as promising therapeutic agents for AMD-associated immune dysregulation.

## 4. Conclusions and Future Directions

With the global population aging, AMD prevalence continues to rise, positioning it as a critical worldwide public health challenge necessitating focused prevention and treatment strategies. While current clinical therapies demonstrate partial efficacy, they remain constrained by significant limitations. ART and its derivatives have emerged as promising therapeutic candidates for AMD management in recent years. This article reviews the current research progress on ART and its derivatives in AMD treatment.

Extensive studies reveal that ART and its derivatives exert multifaceted biological activities against key AMD pathological mechanisms, encompassing inflammation, angiogenesis, oxidative stress, tissue fibrosis, mitochondrial dysfunction, lipid metabolism dysregulation, and autoimmune responses. These therapeutic effects operate through both direct molecular interactions and indirect modulation of cellular signaling pathways, collectively contributing to retinal lesion repair and AMD progression delay. The multi-target, multi-pathway pharmacological profile not only enables therapeutic synergistic effects but also minimizes resistance development risk and systemic toxicity, thereby improving overall treatment outcomes. Accumulating clinical evidence supports the safety profile and therapeutic potential of ART and its derivatives. Notably, given the inherent challenges of posterior ocular drug delivery characterized by limited tissue exposure and poor bioavailability, integration with nanoparticle-based delivery platforms appears crucial for enhancing drug permeability, stability, and target specificity [16,185].

Priority research directions should address: First, elucidating spatiotemporal distribution profiles of ART metabolites using advanced metabolomics approaches to establish dynamic dose–response relationships. Second, developing prodrug strategies featuring stabilized peroxide bridges and targeted nanoformulations to optimize therapeutic indices while minimizing off-target effects. Third, comprehensive evaluation of long-term intraocular administration safety, particularly subchronic toxicological effects on photoreceptors and retinal pigment epithelium. Furthermore, clinical trials should incorporate multimodal imaging and functional assessments (e.g., multifocal electroretinography, microperimetry) for dynamic monitoring of retinal function modifications. Although current ART-based AMD research remains in the exploratory phase, synergistic integration of innovative biotechnologies with rational drug design may overcome conventional therapy limitations and provide AMD patients with safer, more sustainable treatment alternatives.

## Figures and Tables

**Table 1:** Effects of artemisinin and its derivatives on AMD.

Artemisinins	Vivo or Vitro	Model	Dosage and Duration	Administration Route	Curative Effects	Potential Mechanism	References
Dihydroartemisinin	in vivo (mice)	laser-induced CNV	200 mg/kg/d for 12 days	Oral (intragastric administration)	Inhibited CNV formation	Suppresses the classic NF-κB signaling pathway and downregulates the expression of VEGFR-2 and VEGF	[48]
Artemisinin	in vitro	CAM assay	Artemisinin 0.025% and dexamethasone 0.025% for 8 h	-	Anti-angiogenic	Produces remarkable good anti-angiogenic effect by its improved solubility and enhanced permeability	[49]
Artemisinin (loaded nanomicelles)	in vitro	CAM assay	Artemisinin 0.05% for 24 h	-	Anti-angiogenic	Increases solubility, promotes corneal penetration and affects drug release	[16]
Artesunate	in vitro/ in vivo (mice)	ChEC cell/ laser-induced CNV	10 μM for 24 h/ 8 μg (4 μg per dose, once weekly)	-/ Intravitreal injection	Inhibited CNV and the accompanying fibrosis	Reduces inflammatory factors, downregulates fibrotic factors and inhibits MP recruitment	[47]
Artesunate	in vivo (rabbit/ monkey)	ocular neovascularization	A single dose of 1 μg/ A single dose of 20 μg	intravitreal injection	Attenuated ocular neovascularization and macular edema	Downregulates VEGFR-2, PKCα, and PDGFR expression	[50]
Artemisinin	in vitro	D407 cells	3–100 μM for 2 h	-	Reduce oxidative stress	Inhibits the generation of intracellular ROS, modulates △ψm and caspase 3/7 dependent pathway, and activates ERK1/2 signaling	[17]
Artemisinin	in vitro	D407 and primary cultured RPE cells	3.125–100 μM for 2 h	-	Reduce oxidative stress	Reduces intracellular ROS generation and oxidative stress, decreases LDH release and the loss of mitochondrial membrane potential, and enhances the activation of AMPK	[51]
Artemisinin	in vitro	D407 and ARPE19 cell line	20 μM for 1 h	-	Reduce oxidative stress	Increase Acetyl-H4 (Lys 8) level	[52]
Artemisinin	in vitro/ in vivo (SD rats)	RGC-5 cells/ light-exposed retinal damage	6.25–100 μM for 24 h/ 30, 100, 300 μg/mL	-/ Intravitreous injection	Inhibit oxidative damage	Decreases the production of intracellular ROS, increases mitochondrial membrane potential, decreases cell apoptosis and upregulates the phosphorylation of p38 and ERK1/2	[53]

**Table 2:** Mitochondrial dysregulation in AMD pathogenesis and therapeutic modulation by ART and its derivatives.

Mitochondrial Process	Role in AMD Pathogenesis	ART’s Therapeutic Action	References
Energy production	ROS induces RPE oxidative damage	Inhibits ROS via ERK1/2 and p38 signaling pathways	[53]
Fusion-fission dynamics	Fragmentation impairs metabolic efficiency	Enhances fusion kinetics and delays fragmentation	[144]
Membrane potential stability	Loss of ΔΨm triggers apoptosis	Activates AMPK to stabilize ΔΨm and inhibit caspase-3	[51,143]
Biosynthesis (PGC-1α)	Impaired biosynthesis reduces antioxidant capacity	Upregulates PGC-1α and regulates pyruvate metabolism	[145,146,147]
Redox homeostasis	Oxidative imbalance disrupts Bruch’s membrane	Restores balance via H_2_O_2_ regulation and Nrf2 activation	[109,148]

## Abbreviations

The following abbreviations are used in this manuscript:

AMD	Age-related macular degeneration
VEGF	Vascular endothelial growth factor
ART	Artemisinin
CNV	Choroidal neovascularization
ARTS	Artesunate
DHA	Dihydroartemisinin
TNF-α	Tumor necrosis factor-α
IFNγ	Interferon γ
IL-1β	Interleukin-1β
NF-κB	Nuclear factor-κB
ROS	Reactive oxygen species
AMPK	Adenosine monophosphate-activated protein kinase
HDL	High-density lipoproteins
LDL	Low-density lipoproteins
TG	Triglycerides
TC	Total cholesterol

## References

[1] Wong W.L., Su X., Li X., Cheung C.M., Klein R., Cheng C.Y., Wong T.Y. (2014). Global prevalence of age-related macular degeneration and disease burden projection for 2020 and 2040: A systematic review and meta-analysis. Lancet Glob. Health.

[2] Jonas J.B., Cheung C.M.G., Panda-Jonas S. (2017). Updates on the Epidemiology of Age-Related Macular Degeneration. Asia-Pac. J. Ophthalmol..

[3] Chapman N.A., Jacobs R.J., Braakhuis A.J. (2019). Role of diet and food intake in age-related macular degeneration: A systematic review. Clin. Exp. Ophthalmol..

[4] Hadziahmetovic M., Malek G. (2020). Age-Related Macular Degeneration Revisited: From Pathology and Cellular Stress to Potential Therapies. Front. Cell Dev. Biol..

[5] Blasiak J., Chojnacki J., Szczepanska J., Fila M., Chojnacki C., Kaarniranta K., Pawlowska E. (2023). Epigallocatechin-3-Gallate, an Active Green Tea Component to Support Anti-VEGFA Therapy in Wet Age-Related Macular Degeneration. Nutrients.

[6] Deng Y., Qiao L., Du M., Qu C., Wan L., Li J., Huang L. (2022). Age-related macular degeneration: Epidemiology, genetics, pathophysiology, diagnosis, and targeted therapy. Genes Dis..

[7] Apte R.S. (2021). Age-Related Macular Degeneration. N. Engl. J. Med..

[8] Kaiser S.M., Arepalli S., Ehlers J.P. (2021). Current and Future Anti-VEGF Agents for Neovascular Age-Related Macular Degeneration. J. Exp. Pharmacol..

[9] Song D., Liu P., Shang K., Ma Y. (2022). Application and mechanism of anti-VEGF drugs in age-related macular degeneration. Front. Bioeng. Biotechnol..

[10] Nguyen Q.D., Das A., Do D.V., Dugel P.U., Gomes A., Holz F.G., Koh A., Pan C.K., Sepah Y.J., Patel N. (2020). Brolucizumab: Evolution through Preclinical and Clinical Studies and the Implications for the Management of Neovascular Age-Related Macular Degeneration. Ophthalmology.

[11] Casalino G., Stevenson M.R., Bandello F., Chakravarthy U. (2018). Tomographic Biomarkers Predicting Progression to Fibrosis in Treated Neovascular Age-Related Macular Degeneration: A Multimodal Imaging Study. Ophthalmol. Retin..

[12] Khoramnia R., Figueroa M.S., Hattenbach L.O., Pavesio C.E., Anderesi M., Schmouder R., Chen Y., de Smet M.D. (2022). Manifestations of intraocular inflammation over time in patients on brolucizumab for neovascular AMD. Graefe Arch. Clin. Exp. Ophthalmol. = Albrecht Von Graefes Arch. Klin. Exp. Ophthalmol..

[13] Posadino A.M., Giordo R., Pintus G., Mohammed S.A., Orhan I.E., Fokou P.V.T., Sharopov F., Adetunji C.O., Gulsunoglu-Konuskan Z., Ydyrys A. (2023). Medicinal and mechanistic overview of artemisinin in the treatment of human diseases. Biomed. Pharmacother. = Biomed. Pharmacother..

[14] Nabi N., Singh S., Saffeullah P. (2023). An updated review on distribution, biosynthesis and pharmacological effects of artemisinin: A wonder drug. Phytochemistry.

[15] Shi Q., Xia F., Wang Q., Liao F., Guo Q., Xu C., Wang J. (2022). Discovery and repurposing of artemisinin. Front. Med..

[16] Ponnusamy C., Sugumaran A., Krishnaswami V., Palanichamy R., Velayutham R., Natesan S. (2021). Development and Evaluation of Polyvinylpyrrolidone K90 and Poloxamer 407 Self-Assembled Nanomicelles: Enhanced Topical Ocular Delivery of Artemisinin. Polymers.

[17] Chong C.M., Zheng W. (2016). Artemisinin protects human retinal pigment epithelial cells from hydrogen peroxide-induced oxidative damage through activation of ERK/CREB signaling. Redox Biol..

[18] Tu Y. (2016). Artemisinin-A Gift from Traditional Chinese Medicine to the World (Nobel Lecture). Angew. Chem..

[19] Navaratnam V., Mansor S.M., Sit N.W., Grace J., Li Q., Olliaro P. (2000). Pharmacokinetics of artemisinin-type compounds. Clin. Pharmacokinet..

[20] Kong L.Y., Tan R.X. (2015). Artemisinin, a miracle of traditional Chinese medicine. Nat. Prod. Rep..

[21] Jin Q., Liu T., Chen D., Yang L., Mao H., Ma F., Wang Y., Li P., Zhan Y. (2023). Therapeutic potential of artemisinin and its derivatives in managing kidney diseases. Front. Pharmacol..

[22] Tsui K.H., Wu M.Y., Lin L.T., Wen Z.H., Li Y.H., Chu P.Y., Li C.J. (2019). Disruption of mitochondrial homeostasis with artemisinin unravels anti-angiogenesis effects via auto-paracrine mechanisms. Theranostics.

[23] Fu W., Ma Y., Li L., Liu J., Fu L., Guo Y., Zhang Z., Li J., Jiang H. (2020). Artemether Regulates Metaflammation to Improve Glycolipid Metabolism in db/db Mice. Diabetes Metab. Syndr. Obes. Targets Ther..

[24] Dolivo D., Weathers P., Dominko T. (2021). Artemisinin and artemisinin derivatives as anti-fibrotic therapeutics. Acta Pharm. Sin. B.

[25] Lu X., Efferth T. (2021). Repurposing of artemisinin-type drugs for the treatment of acute leukemia. Semin. Cancer Biol..

[26] Xie K., Li Z., Zhang Y., Wu H., Zhang T., Wang W. (2024). Artemisinin and its derivatives as promising therapies for autoimmune diseases. Heliyon.

[27] Huang Y., Yang Y., Liu G., Xu M. (2023). New clinical application prospects of artemisinin and its derivatives: A scoping review. Infect. Dis. Poverty.

[28] Tarning J., Hanboonkunupakarn B., Hoglund R.M., Chotivanich K., Mukaka M., Pukrittayakamee S., Day N.P.J., White N.J., Dondorp A.M., Jittamala P. (2024). Safety and pharmacokinetic properties of a new formulation of parenteral artesunate in healthy Thai volunteers. Malar. J..

[29] Sun C., Cao Y., Zhu P., Zhou B. (2017). A mitochondria-targeting artemisinin derivative with sharply increased antitumor but depressed anti-yeast and anti-malaria activities. Sci. Rep..

[30] Aquino I., Perazzo F.F., Maistro E.L. (2011). Genotoxicity assessment of the antimalarial compound artesunate in somatic cells of mice. Food Chem. Toxicol. Int. J. Publ. Br. Ind. Biol. Res. Assoc..

[31] Sun C., Zhou B. (2017). The antimalarial drug artemisinin induces an additional, Sod1-supressible anti-mitochondrial action in yeast. Biochim. Biophys. Acta Mol. Cell Res..

[32] Trincão-Marques J., Ayton L.N., Hickey D.G., Marques-Neves C., Guymer R.H., Edwards T.L., Sousa D.C. (2024). Gene and cell therapy for age-related macular degeneration: A review. Surv. Ophthalmol..

[33] Bhumika, Bora N.S., Bora P.S. (2024). Genetic Insights into Age-Related Macular Degeneration. Biomedicines.

[34] Corydon T.J., Bek T. (2025). Multiple gene therapy as a tool for regulating the expression of molecules involved in neovascular age-related macular degeneration. Prog. Retin. Eye Res..

[35] Heesterbeek T.J., Lorés-Motta L., Hoyng C.B., Lechanteur Y.T.E., den Hollander A.I. (2020). Risk factors for progression of age-related macular degeneration. Ophthalmic Physiol. Opt. J. Br. Coll. Ophthalmic Opt..

[36] Chakravarthy U., Wong T.Y., Fletcher A., Piault E., Evans C., Zlateva G., Buggage R., Pleil A., Mitchell P. (2010). Clinical risk factors for age-related macular degeneration: A systematic review and meta-analysis. BMC Ophthalmol..

[37] Lambert N.G., ElShelmani H., Singh M.K., Mansergh F.C., Wride M.A., Padilla M., Keegan D., Hogg R.E., Ambati B.K. (2016). Risk factors and biomarkers of age-related macular degeneration. Prog. Retin. Eye Res..

[38] Ruan Y., Jiang S., Gericke A. (2021). Age-Related Macular Degeneration: Role of Oxidative Stress and Blood Vessels. Int. J. Mol. Sci..

[39] Park Y.G., Park Y.S., Kim I.B. (2021). Complement System and Potential Therapeutics in Age-Related Macular Degeneration. Int. J. Mol. Sci..

[40] Manikandan S.K., Logan A., Cerrada-Gimenez M., Fitzhenry L., Coffey L., Kaja S., Rani S. (2023). Immune System, Inflammation and Autoantigens in Wet Age-Related Macular Degeneration: Pathological Significance and Therapeutic Importance. Life.

[41] Terluk M.R., Ebeling M.C., Fisher C.R., Kapphahn R.J., Yuan C., Kartha R.V., Montezuma S.R., Ferrington D.A. (2019). N-Acetyl-L-cysteine Protects Human Retinal Pigment Epithelial Cells from Oxidative Damage: Implications for Age-Related Macular Degeneration. Oxidative Med. Cell. Longev..

[42] Zhao H., Wang R., Ye M., Zhang L. (2019). Genipin protects against H_2_O_2_-induced oxidative damage in retinal pigment epithelial cells by promoting Nrf2 signaling. Int. J. Mol. Med..

[43] Ding X., Patel M., Chan C.C. (2009). Molecular pathology of age-related macular degeneration. Prog. Retin. Eye Res..

[44] Edwards M., Lutty G.A. (2021). Bruch’s Membrane and the Choroid in Age-Related Macular Degeneration. Adv. Exp. Med. Biol..

[45] Curcio C.A., Zanzottera E.C., Ach T., Balaratnasingam C., Freund K.B. (2017). Activated Retinal Pigment Epithelium, an Optical Coherence Tomography Biomarker for Progression in Age-Related Macular Degeneration. Investig. Ophthalmol. Vis. Sci..

[46] Wong T.Y., Chakravarthy U., Klein R., Mitchell P., Zlateva G., Buggage R., Fahrbach K., Probst C., Sledge I. (2008). The natural history and prognosis of neovascular age-related macular degeneration: A systematic review of the literature and meta-analysis. Ophthalmology.

[47] Sheibani N., Song Y.S., Farnoodian M., Inampudi S., Wang S., Darjatmoko S.R., Sorenson C.M. (2023). Artesunate mitigates choroidal neovascularization and scar formation. Exp. Eye Res..

[48] Li X., Gao S., Zhang Y., Xin M., Zuo C., Yan N., Xia Q., Zhang M. (2022). Dihydroartemisinin Inhibits Laser-Induced Choroidal Neovascularization in a Mouse Model of Neovascular AMD. Front. Pharmacol..

[49] Ponnusamy C., Sugumaran A., Krishnaswami V., Kandasamy R., Natesan S. (2019). Design and development of artemisinin and dexamethasone loaded topical nanodispersion for the effective treatment of age-related macular degeneration. IET Nanobiotechnol..

[50] Zong Y., Yuan Y., Qian X., Huang Z., Yang W., Lin L., Zheng Q., Li Y., He H., Gao Q. (2016). Small Molecular-Sized Artesunate Attenuates Ocular Neovascularization via VEGFR2, PKCα, and PDGFR Targets. Sci. Rep..

[51] Li S., Chaudhary S.C., Zhao X., Gaur U., Fang J., Yan F., Zheng W. (2019). Artemisinin Protects Human Retinal Pigmented Epithelial Cells Against Hydrogen Peroxide-induced Oxidative Damage by Enhancing the Activation of AMP-active Protein Kinase. Int. J. Biol. Sci..

[52] Yang C., Ge L., Yu X., Lazarovici P., Zheng W. (2024). Artemisinin Confers Cytoprotection toward Hydrogen Peroxide-Induced Cell Apoptosis in Retinal Pigment Epithelial Cells in Correlation with the Increased Acetylation of Histone H4 at Lysine 8. Molecules.

[53] Yan F., Wang H., Gao Y., Xu J., Zheng W. (2017). Artemisinin Protects Retinal Neuronal Cells against Oxidative Stress and Restores Rat Retinal Physiological Function from Light Exposed Damage. ACS Chem. Neurosci..

[54] Cheng S.C., Huang W.C., JH S.P., Wu Y.H., Cheng C.Y. (2019). Quercetin Inhibits the Production of IL-1β-Induced Inflammatory Cytokines and Chemokines in ARPE-19 Cells via the MAPK and NF-κB Signaling Pathways. Int. J. Mol. Sci..

[55] Leung K.W., Barnstable C.J., Tombran-Tink J. (2009). Bacterial endotoxin activates retinal pigment epithelial cells and induces their degeneration through IL-6 and IL-8 autocrine signaling. Mol. Immunol..

[56] Krogh Nielsen M., Subhi Y., Molbech C.R., Falk M.K., Nissen M.H., Sørensen T.L. (2019). Systemic Levels of Interleukin-6 Correlate With Progression Rate of Geographic Atrophy Secondary to Age-Related Macular Degeneration. Investig. Ophthalmol. Vis. Sci..

[57] Coughlin B., Schnabolk G., Joseph K., Raikwar H., Kunchithapautham K., Johnson K., Moore K., Wang Y., Rohrer B. (2016). Connecting the innate and adaptive immune responses in mouse choroidal neovascularization via the anaphylatoxin C5a and γδT-cells. Sci. Rep..

[58] Ishikawa K., Kannan R., Hinton D.R. (2016). Molecular mechanisms of subretinal fibrosis in age-related macular degeneration. Exp. Eye Res..

[59] Pugazhendhi A., Hubbell M., Jairam P., Ambati B. (2021). Neovascular Macular Degeneration: A Review of Etiology, Risk Factors, and Recent Advances in Research and Therapy. Int. J. Mol. Sci..

[60] Miraghazadeh B., Cook M.C. (2018). Nuclear Factor-kappaB in Autoimmunity: Man and Mouse. Front. Immunol..

[61] Wang H., Chen Y., Han J., Meng Q., Xi Q., Wu G., Zhang B. (2016). DCAF4L2 promotes colorectal cancer invasion and metastasis via mediating degradation of NFκb negative regulator PPM1B. Am. J. Transl. Res..

[62] Sun S.C. (2017). The non-canonical NF-κB pathway in immunity and inflammation. Nat. Rev. Immunol..

[63] Zhao X., Huang X., Yang C., Jiang Y., Zhou W., Zheng W. (2022). Artemisinin Attenuates Amyloid-Induced Brain Inflammation and Memory Impairments by Modulating TLR4/NF-κB Signaling. Int. J. Mol. Sci..

[64] Wang Y., Huang Z., Wang L., Meng S., Fan Y., Chen T., Cao J., Jiang R., Wang C. (2011). The anti-malarial artemisinin inhibits pro-inflammatory cytokines via the NF-κB canonical signaling pathway in PMA-induced THP-1 monocytes. Int. J. Mol. Med..

[65] Cao Q., Jiang Y., Shi J., Xu C., Liu X., Yang T., Fu P., Niu T. (2015). Artemisinin inhibits the proliferation, migration, and inflammatory reaction induced by tumor necrosis factor-α in vascular smooth muscle cells through nuclear factor kappa B pathway. J. Surg. Res..

[66] Wang D., Shi J., Lv S., Xu W., Li J., Ge W., Xiao C., Geng D., Liu Y. (2015). Artesunate Attenuates Lipopolysaccharide-Stimulated Proinflammatory Responses by Suppressing TLR4, MyD88 Expression, and NF-κB Activation in Microglial Cells. Inflammation.

[67] Liu H., Tian Q., Ai X., Qin Y., Cui Z., Li M., Yang J., Zhai D., Liu Y., Chen S. (2017). Dihydroartemisinin attenuates autoimmune thyroiditis by inhibiting the CXCR3/PI3K/AKT/NF-κB signaling pathway. Oncotarget.

[68] Wang J.X., Hou L.F., Yang Y., Tang W., Li Y., Zuo J.P. (2009). SM905, an artemisinin derivative, inhibited NO and pro-inflammatory cytokine production by suppressing MAPK and NF-kappaB pathways in RAW 264.7 macrophages. Acta Pharmacol. Sin..

[69] Arthur J.S., Ley S.C. (2013). Mitogen-activated protein kinases in innate immunity. Nat. Rev. Immunol..

[70] Muraleva N.A., Kolosova N.G. (2023). P38 MAPK Signaling in the Retina: Effects of Aging and Age-Related Macular Degeneration. Int. J. Mol. Sci..

[71] Wang Y., Ma H., Yang Q., Chen K., Ye H., Wang X., Xia J., Chen X., Wang X., Shen Y. (2025). Senescent retinal pigment epithelial cells promote angiogenesis in choroidal neovascularization via the TAK1/p38 MAPK pathway. Exp. Eye Res..

[72] Zhao X., Liu M., Li J., Yin S., Wu Y., Wang A. (2017). Antimalarial agent artesunate protects Concanavalin A-induced autoimmune hepatitis in mice by inhibiting inflammatory responses. Chem.-Biol. Interact..

[73] Wang Y., Cao J., Fan Y., Xie Y., Xu Z., Yin Z., Gao L., Wang C. (2016). Artemisinin inhibits monocyte adhesion to HUVECs through the NF-κB and MAPK pathways in vitro. Int. J. Mol. Med..

[74] Wang K.S., Li J., Wang Z., Mi C., Ma J., Piao L.X., Xu G.H., Li X., Jin X. (2017). Artemisinin inhibits inflammatory response via regulating NF-κB and MAPK signaling pathways. Immunopharmacol. Immunotoxicol..

[75] Wang F., Gao Q., Yang J., Wang C., Cao J., Sun J., Fan Z., Fu L. (2020). Artemisinin suppresses myocardial ischemia-reperfusion injury via NLRP3 inflammasome mechanism. Mol. Cell. Biochem..

[76] Liang R., Chen W., Fan H., Chen X., Zhang J., Zhu J.S. (2020). Dihydroartemisinin prevents dextran sodium sulphate-induced colitisthrough inhibition of the activation of NLRP3 inflammasome and p38 MAPK signaling. Int. Immunopharmacol..

[77] Yang F.M., Fan D., Yang X.Q., Zhu F.H., Shao M.J., Li Q., Liu Y.T., Lin Z.M., Cao S.Q., Tang W. (2021). The artemisinin analog SM934 alleviates dry eye disease in rodent models by regulating TLR4/NF-κB/NLRP3 signaling. Acta Pharmacol. Sin..

[78] Kanazawa H. (2007). VEGF, angiopoietin-1 and -2 in bronchial asthma: New molecular targets in airway angiogenesis and microvascular remodeling. Recent Pat. Inflamm. Allergy Drug Discov..

[79] Lu M., Adamis A.P. (2006). Molecular biology of choroidal neovascularization. Ophthalmol. Clin. N. Am..

[80] Fantin A., Vieira J.M., Gestri G., Denti L., Schwarz Q., Prykhozhij S., Peri F., Wilson S.W., Ruhrberg C. (2010). Tissue macrophages act as cellular chaperones for vascular anastomosis downstream of VEGF-mediated endothelial tip cell induction. Blood.

[81] Meng L.B., Zhang Y.M., Shan M.J., Qiu Y., Zhang T.J., Gong T. (2019). Pivotal micro factors associated with endothelial cells. Chin. Med. J..

[82] Ferrara N. (2004). Vascular endothelial growth factor: Basic science and clinical progress. Endocr. Rev..

[83] Abou-Khalil R., Mounier R., Chazaud B. (2010). Regulation of myogenic stem cell behavior by vessel cells: The “ménage à trois” of satellite cells, periendothelial cells and endothelial cells. Cell Cycle.

[84] Chen Q., Liu Y., Jeong H.W., Stehling M., Dinh V.V., Zhou B., Adams R.H. (2019). Apelin(+) Endothelial Niche Cells Control Hematopoiesis and Mediate Vascular Regeneration after Myeloablative Injury. Cell Stem Cell.

[85] Sack K.D., Teran M., Nugent M.A. (2016). Extracellular Matrix Stiffness Controls VEGF Signaling and Processing in Endothelial Cells. J. Cell. Physiol..

[86] Russo T.A., Banuth A.M.M., Nader H.B., Dreyfuss J.L. (2020). Altered shear stress on endothelial cells leads to remodeling of extracellular matrix and induction of angiogenesis. PLoS ONE.

[87] Riddell J.R., Maier P., Sass S.N., Moser M.T., Foster B.A., Gollnick S.O. (2012). Peroxiredoxin 1 stimulates endothelial cell expression of VEGF via TLR4 dependent activation of HIF-1α. PLoS ONE.

[88] Zhu X.X., Yang L., Li Y.J., Zhang D., Chen Y., Kostecká P., Kmoníčková E., Zídek Z. (2013). Effects of sesquiterpene, flavonoid and coumarin types of compounds from *Artemisia annua* L. on production of mediators of angiogenesis. Pharmacol. Rep. PR.

[89] Chen H.H., Zhou H.J., Wu G.D., Lou X.E. (2004). Inhibitory effects of artesunate on angiogenesis and on expressions of vascular endothelial growth factor and VEGF receptor KDR/flk-1. Pharmacology.

[90] Verma S., Das P., Kumar V.L. (2017). Chemoprevention by artesunate in a preclinical model of colorectal cancer involves down regulation of β-catenin, suppression of angiogenesis, cellular proliferation and induction of apoptosis. Chem.-Biol. Interact..

[91] Guo L., Dong F., Hou Y., Cai W., Zhou X., Huang A.L., Yang M., Allen T.D., Liu J. (2014). Dihydroartemisinin inhibits vascular endothelial growth factor-induced endothelial cell migration by a p38 mitogen-activated protein kinase-independent pathway. Exp. Ther. Med..

[92] Dong F., Zhou X., Li C., Yan S., Deng X., Cao Z., Li L., Tang B., Allen T.D., Liu J. (2014). Dihydroartemisinin targets VEGFR2 via the NF-κB pathway in endothelial cells to inhibit angiogenesis. Cancer Biol. Ther..

[93] Li Y., Xiao X., Wang H., Zhou Q., Jin Z., Zhang Y., Wang Y., Yue F., Zhou S., Yang J. (2021). Integrating network pharmacology and experimental models to investigate the mechanisms of dihydroartemisinin in preventing NSCLC progression via mTOR/HIF-1α signaling. Eur. J. Pharmacol..

[94] Chen H.H., Zhou H.J., Fang X. (2003). Inhibition of human cancer cell line growth and human umbilical vein endothelial cell angiogenesis by artemisinin derivatives in vitro. Pharmacol. Res..

[95] Grassi M.O., Monteleone G., Pozharitskiy N., Molfetta T., Boscia G., Alessio G., Boscia F. (2023). SEVERE VISUAL LOSS DURING ANTI-VEGF INTRAVITREAL INJECTIONS IN NEOVASCULAR AGE-RELATED MACULAR DEGENERATION: TIMING, PROGNOSIS, AND OPTICAL COHERENCE TOMOGRAPHY FINDINGS. Retina.

[96] Nichani P.A.H., Popovic M.M., Dhoot A.S., Pathak A., Muni R.H., Kertes P.J. (2023). Treat-and-extend dosing of intravitreal anti-VEGF agents in neovascular age-related macular degeneration: A meta-analysis. Eye.

[97] Yu D.Y., Cringle S.J. (2005). Retinal degeneration and local oxygen metabolism. Exp. Eye Res..

[98] Ray P.D., Huang B.W., Tsuji Y. (2012). Reactive oxygen species (ROS) homeostasis and redox regulation in cellular signaling. Cell. Signal..

[99] Beatty S., Koh H., Phil M., Henson D., Boulton M. (2000). The role of oxidative stress in the pathogenesis of age-related macular degeneration. Surv. Ophthalmol..

[100] Yu D.Y., Cringle S.J. (2001). Oxygen distribution and consumption within the retina in vascularised and avascular retinas and in animal models of retinal disease. Prog. Retin. Eye Res..

[101] Chan C.M., Huang D.Y., Sekar P., Hsu S.H., Lin W.W. (2019). Reactive oxygen species-dependent mitochondrial dynamics and autophagy confer protective effects in retinal pigment epithelial cells against sodium iodate-induced cell death. J. Biomed. Sci..

[102] Tokarz P., Kaarniranta K., Blasiak J. (2013). Role of antioxidant enzymes and small molecular weight antioxidants in the pathogenesis of age-related macular degeneration (AMD). Biogerontology.

[103] Mitter S.K., Rao H.V., Qi X., Cai J., Sugrue A., Dunn W.A., Grant M.B., Boulton M.E. (2012). Autophagy in the retina: A potential role in age-related macular degeneration. Adv. Exp. Med. Biol..

[104] Wu D.M., Ji X., Ivanchenko M.V., Chung M., Piper M., Rana P., Wang S.K., Xue Y., West E., Zhao S.R. (2021). Nrf2 overexpression rescues the RPE in mouse models of retinitis pigmentosa. JCI Insight.

[105] Mitter S.K., Song C., Qi X., Mao H., Rao H., Akin D., Lewin A., Grant M., Dunn W., Ding J. (2014). Dysregulated autophagy in the RPE is associated with increased susceptibility to oxidative stress and AMD. Autophagy.

[106] Chae J.B., Jang H., Son C., Park C.W., Choi H., Jin S., Lee H.Y., Lee H., Ryu J.H., Kim N. (2021). Targeting senescent retinal pigment epithelial cells facilitates retinal regeneration in mouse models of age-related macular degeneration. GeroScience.

[107] Brown E.E., DeWeerd A.J., Ildefonso C.J., Lewin A.S., Ash J.D. (2019). Mitochondrial oxidative stress in the retinal pigment epithelium (RPE) led to metabolic dysfunction in both the RPE and retinal photoreceptors. Redox Biol..

[108] Hytti M., Piippo N., Salminen A., Honkakoski P., Kaarniranta K., Kauppinen A. (2015). Quercetin alleviates 4-hydroxynonenal-induced cytotoxicity and inflammation in ARPE-19 cells. Exp. Eye Res..

[109] Yang C., Zhao X., Zhou W., Li Q., Lazarovici P., Zheng W. (2024). Artemisinin conferred cytoprotection to human retinal pigment epithelial cells exposed to amiodarone-induced oxidative insult by activating the CaMKK2/AMPK/Nrf2 pathway. J. Transl. Med..

[110] Kim W.S., Choi W.J., Lee S., Kim W.J., Lee D.C., Sohn U.D., Shin H.S., Kim W. (2015). Anti-inflammatory, Antioxidant and Antimicrobial Effects of Artemisinin Extracts from *Artemisia annua* L. Korean J. Physiol. Pharmacol. Off. J. Korean Physiol. Soc. Korean Soc. Pharmacol..

[111] Guruprasad B., Chaudhary P., Choedon T., Kumar V.L. (2015). Artesunate Ameliorates Functional Limitations in Freund’s Complete Adjuvant-Induced Monoarthritis in Rat by Maintaining Oxidative Homeostasis and Inhibiting COX-2 Expression. Inflammation.

[112] Wohlfahrt T., Rauber S., Uebe S., Luber M., Soare A., Ekici A., Weber S., Matei A.E., Chen C.W., Maier C. (2019). PU.1 controls fibroblast polarization and tissue fibrosis. Nature.

[113] Juillerat-Jeanneret L., Aubert J.D., Mikulic J., Golshayan D. (2018). Fibrogenic Disorders in Human Diseases: From Inflammation to Organ Dysfunction. J. Med. Chem..

[114] Cheong K.X., Cheung C.M.G., Teo K.Y.C. (2023). Review of Fibrosis in Neovascular Age-Related Macular Degeneration. Am. J. Ophthalmol..

[115] Kalluri R., Weinberg R.A. (2009). The basics of epithelial-mesenchymal transition. J. Clin. Investig..

[116] Little K., Llorián-Salvador M., Tang M., Du X., Marry S., Chen M., Xu H. (2020). Macrophage to myofibroblast transition contributes to subretinal fibrosis secondary to neovascular age-related macular degeneration. J. Neuroinflamm..

[117] Patel P.J., Villavicencio P., Hanumunthadu D. (2023). Systematic Review of Neovascular Age-Related Macular Degeneration Disease Activity Criteria Use to Shorten, Maintain or Extend Treatment Intervals with Anti-VEGF in Clinical Trials: Implications for Clinical Practice. Ophthalmol. Ther..

[118] Chandra S., Arpa C., Menon D., Khalid H., Hamilton R., Nicholson L., Pal B., Fasolo S., Hykin P., Keane P.A. (2020). Ten-year outcomes of antivascular endothelial growth factor therapy in neovascular age-related macular degeneration. Eye.

[119] Roberts P.K., Zotter S., Montuoro A., Pircher M., Baumann B., Ritter M., Hitzenberger C.K., Schmidt-Erfurth U. (2019). Identification and Quantification of the Angiofibrotic Switch in Neovascular AMD. Investig. Ophthalmol. Vis. Sci..

[120] Wang Y., Wang Y., You F., Xue J. (2020). Novel use for old drugs: The emerging role of artemisinin and its derivatives in fibrosis. Pharmacol. Res..

[121] Zhang H., Yang H., Liu X.M., Ying J., Zu T., Jiang J., Liu M.M., Jin J. (2024). Targeted inhibition of transforming growth factor-β type I receptor by AZ12601011 improves paraquat poisoning-induced multiple organ fibrosis. Pestic. Biochem. Physiol..

[122] Lee K.M., Hwang Y.J., Jung G.S. (2024). Alantolactone Attenuates Renal Fibrosis via Inhibition of Transforming Growth Factor β/Smad3 Signaling Pathway. Diabetes Metab. J..

[123] Khalil H., Kanisicak O., Prasad V., Correll R.N., Fu X., Schips T., Vagnozzi R.J., Liu R., Huynh T., Lee S.J. (2017). Fibroblast-specific TGF-β-Smad2/3 signaling underlies cardiac fibrosis. J. Clin. Investig..

[124] Meng X.M., Nikolic-Paterson D.J., Lan H.Y. (2016). TGF-β: The master regulator of fibrosis. Nat. Rev. Nephrol..

[125] Park J.H., Park B., Park K.K. (2017). Suppression of Hepatic Epithelial-to-Mesenchymal Transition by Melittin via Blocking of TGFβ/Smad and MAPK-JNK Signaling Pathways. Toxins.

[126] Zheng Y.J., Li X., Sun L., Guo J.W. (2019). Therapeutic effect of dihydroartemisinin on pulmonary fibrosis in rats with dust. Zhonghua Lao Dong Wei Sheng Zhi Ye Bing Za Zhi = Zhonghua Laodong Weisheng Zhiyebing Zazhi = Chin. J. Ind. Hyg. Occup. Dis..

[127] Nong X., Rajbanshi G., Chen L., Li J., Li Z., Liu T., Chen S., Wei G., Li J. (2019). Effect of artesunate and relation with TGF-β1 and SMAD3 signaling on experimental hypertrophic scar model in rabbit ear. Arch. Dermatol. Res..

[128] Li H.X., Liu H., Wang C.M., Wang H.J., Chen J. (2014). Artesunate restraining MAPK passage by smad7 to resist pulmonary fibrosis. Eur. Rev. Med. Pharmacol. Sci..

[129] Wang Z.Y., Zhang Y., Wu L.D., Chen J., Chen M.L., Chen C.M., Xu Q.H. (2022). Artesunate inhibits proliferation and migration of RPE cells and TGF-β2 mediated epithelial mesenchymal transition by suppressing PI3K/AKT pathway. Int. J. Ophthalmol..

[130] Wang Y., Huang G., Mo B., Wang C. (2016). Artesunate modulates expression of matrix metalloproteinases and their inhibitors as well as collagen-IV to attenuate pulmonary fibrosis in rats. Genet. Mol. Res. GMR.

[131] Xu Y., Liu W., Fang B., Gao S., Yan J. (2014). Artesunate ameliorates hepatic fibrosis induced by bovine serum albumin in rats through regulating matrix metalloproteinases. Eur. J. Pharmacol..

[132] Lv J., Bai R., Wang L., Gao J., Zhang H. (2018). Artesunate may inhibit liver fibrosis via the FAK/Akt/β-catenin pathway in LX-2 cells. BMC Pharmacol. Toxicol..

[133] Liu J., Pan Z., Tong B., Wang C., Yang J., Zou J., Jiang J., Zhang L., Jiang B. (2023). Artesunate protects against ocular fibrosis by suppressing fibroblast activation and inducing mitochondria-dependent ferroptosis. FASEB J. Off. Publ. Fed. Am. Soc. Exp. Biol..

[134] Larson S.A., Dolivo D.M., Dominko T. (2019). Artesunate inhibits myofibroblast formation via induction of apoptosis and antagonism of pro-fibrotic gene expression in human dermal fibroblasts. Cell Biol. Int..

[135] Strauss O. (2005). The retinal pigment epithelium in visual function. Physiol. Rev..

[136] Miceli M.V., Liles M.R., Newsome D.A. (1994). Evaluation of oxidative processes in human pigment epithelial cells associated with retinal outer segment phagocytosis. Exp. Cell Res..

[137] King A., Gottlieb E., Brooks D.G., Murphy M.P., Dunaief J.L. (2004). Mitochondria-derived reactive oxygen species mediate blue light-induced death of retinal pigment epithelial cells. Photochem. Photobiol..

[138] Kaarniranta K., Uusitalo H., Blasiak J., Felszeghy S., Kannan R., Kauppinen A., Salminen A., Sinha D., Ferrington D. (2020). Mechanisms of mitochondrial dysfunction and their impact on age-related macular degeneration. Prog. Retin. Eye Res..

[139] La Cunza N., Tan L.X., Thamban T., Germer C.J., Rathnasamy G., Toops K.A., Lakkaraju A. (2021). Mitochondria-dependent phase separation of disease-relevant proteins drives pathological features of age-related macular degeneration. JCI Insight.

[140] Di Rienzo M., Romagnoli A., Refolo G., Vescovo T., Ciccosanti F., Zuchegna C., Lozzi F., Occhigrossi L., Piacentini M., Fimia G.M. (2024). Role of AMBRA1 in mitophagy regulation: Emerging evidence in aging-related diseases. Autophagy.

[141] Dieguez H.H., Romeo H.E., Alaimo A., Bernal Aguirre N.A., Calanni J.S., Adán Aréan J.S., Alvarez S., Sciurano R., Rosenstein R.E., Dorfman D. (2024). Mitochondrial quality control in non-exudative age-related macular degeneration: From molecular mechanisms to structural and functional recovery. Free Radic. Biol. Med..

[142] Ferrington D.A., Ebeling M.C., Kapphahn R.J., Terluk M.R., Fisher C.R., Polanco J.R., Roehrich H., Leary M.M., Geng Z., Dutton J.R. (2017). Altered bioenergetics and enhanced resistance to oxidative stress in human retinal pigment epithelial cells from donors with age-related macular degeneration. Redox Biol..

[143] Zhao X., Fang J., Li S., Gaur U., Xing X., Wang H., Zheng W. (2019). Artemisinin Attenuated Hydrogen Peroxide H_2_O_2_-Induced Oxidative Injury in SH-SY5Y and Hippocampal Neurons via the Activation of AMPK Pathway. Int. J. Mol. Sci..

[144] Qin Y.R., Ma C.Q., Jiang J.H., Wang D.P., Zhang Q.Q., Liu M.R., Zhao H.R., Fang Q., Liu Y. (2022). Artesunate restores mitochondrial fusion-fission dynamics and alleviates neuronal injury in Alzheimer’s disease models. J. Neurochem..

[145] Cheng X., Zhou P., Weng W., Sun Z., Liu H., Chen Y., Cai Y., Yu X., Wang T., Shao M. (2022). Artemether attenuates renal tubular injury by targeting mitochondria in adriamycin nephropathy mice. Am. J. Transl. Res..

[146] Iacovelli J., Rowe G.C., Khadka A., Diaz-Aguilar D., Spencer C., Arany Z., Saint-Geniez M. (2016). PGC-1α Induces Human RPE Oxidative Metabolism and Antioxidant Capacity. Investig. Ophthalmol. Vis. Sci..

[147] Wang Y., Han P., Wang M., Weng W., Zhan H., Yu X., Yuan C., Shao M., Sun H. (2019). Artemether improves type 1 diabetic kidney disease by regulating mitochondrial function. Am. J. Transl. Res..

[148] Han P., Cai Y., Wang Y., Weng W., Chen Y., Wang M., Zhan H., Yu X., Wang T., Shao M. (2021). Artemether ameliorates kidney injury by restoring redox imbalance and improving mitochondrial function in Adriamycin nephropathy in mice. Sci. Rep..

[149] Schachar I.H., Zahid S., Comer G.M., Stem M., Schachar A.G., Saxe S.J., Gardner T.W., Elner V.M., Jayasundera T. (2013). Quantification of fundus autofluorescence to detect disease severity in nonexudative age-related macular degeneration. JAMA Ophthalmol..

[150] Waldstein S.M., Vogl W.D., Bogunovic H., Sadeghipour A., Riedl S., Schmidt-Erfurth U. (2020). Characterization of Drusen and Hyperreflective Foci as Biomarkers for Disease Progression in Age-Related Macular Degeneration Using Artificial Intelligence in Optical Coherence Tomography. JAMA Ophthalmol..

[151] van Leeuwen E.M., Emri E., Merle B.M.J., Colijn J.M., Kersten E., Cougnard-Gregoire A., Dammeier S., Meester-Smoor M., Pool F.M., de Jong E.K. (2018). A new perspective on lipid research in age-related macular degeneration. Prog. Retin. Eye Res..

[152] Mares-Perlman J.A., Brady W.E., Klein R., VandenLangenberg G.M., Klein B.E., Palta M. (1995). Dietary fat and age-related maculopathy. Arch. Ophthalmol..

[153] Jun S., Datta S., Wang L., Pegany R., Cano M., Handa J.T. (2019). The impact of lipids, lipid oxidation, and inflammation on AMD, and the potential role of miRNAs on lipid metabolism in the RPE. Exp. Eye Res..

[154] Gabrielle P.H. (2022). Lipid metabolism and retinal diseases. Acta Ophthalmol..

[155] Han G., Wei P., He M., Jia L., Su Q., Yang X., Hao R. (2024). Role of plasma fatty acid in age-related macular degeneration: Insights from a mendelian randomization analysis. Lipids Health Dis..

[156] Chong E.W., Kreis A.J., Wong T.Y., Simpson J.A., Guymer R.H. (2008). Dietary omega-3 fatty acid and fish intake in the primary prevention of age-related macular degeneration: A systematic review and meta-analysis. Arch. Ophthalmol..

[157] Wang Y., Wang M., Zhang X., Zhang Q., Nie J., Zhang M., Liu X., Ma L. (2016). The Association between the Lipids Levels in Blood and Risk of Age-Related Macular Degeneration. Nutrients.

[158] Colijn J.M., den Hollander A.I., Demirkan A., Cougnard-Grégoire A., Verzijden T., Kersten E., Meester-Smoor M.A., Merle B.M.J., Papageorgiou G., Ahmad S. (2019). Increased High-Density Lipoprotein Levels Associated with Age-Related Macular Degeneration: Evidence from the EYE-RISK and European Eye Epidemiology Consortia. Ophthalmology.

[159] Landowski M., Bowes Rickman C. (2022). Targeting Lipid Metabolism for the Treatment of Age-Related Macular Degeneration: Insights from Preclinical Mouse Models. J. Ocul. Pharmacol. Ther. Off. J. Assoc. Ocul. Pharmacol. Ther..

[160] Jang B.C. (2016). Artesunate inhibits adipogeneis in 3T3-L1 preadipocytes by reducing the expression and/or phosphorylation levels of C/EBP-α, PPAR-γ, FAS, perilipin A, and STAT-3. Biochem. Biophys. Res. Commun..

[161] Zhang G., Li N., Tong Y., Li P., Han H., Song Q., Yang B., Cui L. (2021). Artemisinin derivatives inhibit adipogenic differentiation of 3T3-L1 preadipocytes through upregulation of CHOP. Biochem. Biophys. Res. Commun..

[162] Guo Y., Cheng Y., Li H., Guan H., Xiao H., Li Y. (2023). The Potential of Artemisinins as Novel Treatment for Thyroid Eye Disease by Inhibiting Adipogenesis in Orbital Fibroblasts. Investig. Ophthalmol. Vis. Sci..

[163] He L.H., Gao J.H., Yu X.H., Wen F.J., Luo J.J., Qin Y.S., Chen M.X., Zhang D.W., Wang Z.B., Tang C.K. (2020). Artesunate inhibits atherosclerosis by upregulating vascular smooth muscle cells-derived LPL expression via the KLF2/NRF2/TCF7L2 pathway. Eur. J. Pharmacol..

[164] Pu S., Liu Y., Liang S., Liu P., Qian H., Wu Q., Wang Y. (2020). The Metabolic Changes of Artesunate and Ursolic Acid on Syrian Golden Hamsters Fed with the High-Fat Diet. Molecules.

[165] Liu P., Wang Y., Tian K., Bai X., Wang Y., Wang Y. (2024). Artesunate inhibits macrophage-like phenotype switching of vascular smooth muscle cells and attenuates vascular inflammatory injury in atherosclerosis via NLRP3. Biomed. Pharmacother. = Biomed. Pharmacother..

[166] Fu W., Hongwei J., Li J. (2023). Artemether treatment improves islet function and metabolic homeostasis in diabetic nonhuman primates. J. Diabetes.

[167] Lei Z., Wu H., Yang Y., Hu Q., Lei Y., Liu W., Nie Y., Yang L., Zhang X., Yang C. (2022). Dihydroartemisinin improves hypercholesterolemia in ovariectomized mice via enhancing vectorial transport of cholesterol and bile acids from blood to bile. Bioorg. Med. Chem..

[168] Cunha-Vaz J.G. (2004). The blood-retinal barriers system. Basic concepts and clinical evaluation. Exp. Eye Res..

[169] Caspi R.R. (2006). Ocular autoimmunity: The price of privilege?. Immunol. Rev..

[170] Allingham M.J., Loksztejn A., Cousins S.W., Mettu P.S. (2021). Immunological Aspects of Age-Related Macular Degeneration. Adv. Exp. Med. Biol..

[171] Moir J., Hyman M.J., Wang J., Shah A., Maatouk C., Flores A., Skondra D. (2023). Associations Between Autoimmune Disease and the Development of Age-Related Macular Degeneration. Investig. Ophthalmol. Vis. Sci..

[172] Morohoshi K., Goodwin A.M., Ohbayashi M., Ono S.J. (2009). Autoimmunity in retinal degeneration: Autoimmune retinopathy and age-related macular degeneration. J. Autoimmun..

[173] Patel N., Ohbayashi M., Nugent A.K., Ramchand K., Toda M., Chau K.Y., Bunce C., Webster A., Bird A.C., Ono S.J. (2005). Circulating anti-retinal antibodies as immune markers in age-related macular degeneration. Immunology.

[174] Fletcher E.L. (2020). Contribution of microglia and monocytes to the development and progression of age related macular degeneration. Ophthalmic Physiol. Opt. J. Br. Coll. Ophthalmic Opt..

[175] Gu J., Pauer G.J., Yue X., Narendra U., Sturgill G.M., Bena J., Gu X., Peachey N.S., Salomon R.G., Hagstrom S.A. (2009). Assessing susceptibility to age-related macular degeneration with proteomic and genomic biomarkers. Mol. Cell. Proteom. MCP.

[176] Lin J.B., Santeford A., Usmani D., Shah A.V., Ruzycki P.A., Apte R.S. (2024). Cell-specific Systemic Immune Signatures Associated with Treatment Burden in Neovascular Age-related Macular Degeneration. Ophthalmol. Sci..

[177] Shakir L., Hussain M., Javeed A., Ashraf M., Riaz A. (2011). Artemisinins and immune system. Eur. J. Pharmacol..

[178] Morad H.O.J., Luqman S., Pinto L.G., Cunningham K.P., Vilar B., Clayton G., Shankar-Hari M., McNaughton P.A. (2022). Artemisinin inhibits neutrophil and macrophage chemotaxis, cytokine production and NET release. Sci. Rep..

[179] Chen Y., Tao T., Wang W., Yang B., Cha X. (2021). Dihydroartemisinin attenuated the symptoms of mice model of systemic lupus erythematosus by restoring the Treg/Th17 balance. Clin. Exp. Pharmacol. Physiol..

[180] Li T., Chen H., Yang Z., Liu X.G., Zhang L.M., Wang H. (2013). Evaluation of the immunosuppressive activity of artesunate in vitro and in vivo. Int. Immunopharmacol..

[181] Shi X., Liao T., Chen Y., Chen J., Liu Y., Zhao J., Dang J., Sun Q., Pan Y. (2024). Dihydroartemisinin inhibits follicular helper T and B cells: Implications for systemic lupus erythematosus treatment. Arch. Pharmacal Res..

[182] Tong X., Chen L., He S.J., Zuo J.P. (2022). Artemisinin derivative SM934 in the treatment of autoimmune and inflammatory diseases: Therapeutic effects and molecular mechanisms. Acta Pharmacol. Sin..

[183] Lin Z.M., Yang X.Q., Zhu F.H., He S.J., Tang W., Zuo J.P. (2016). Artemisinin analogue SM934 attenuate collagen-induced arthritis by suppressing T follicular helper cells and T helper 17 cells. Sci. Rep..

[184] Chen K., Tang L., Nong X. (2023). Artesunate targets cellular metabolism to regulate the Th17/Treg cell balance. Inflamm. Res. Off. J. Eur. Histamine Res. Soc..

[185] Wu Y., Li X., Fu X., Huang X., Zhang S., Zhao N., Ma X., Saiding Q., Yang M., Tao W. (2024). Innovative Nanotechnology in Drug Delivery Systems for Advanced Treatment of Posterior Segment Ocular Diseases. Adv. Sci..

